# Early identification of severe immune checkpoint inhibitor associated myocarditis: From an electrocardiographic perspective

**DOI:** 10.1002/cam4.7460

**Published:** 2024-07-31

**Authors:** Yongyin Gao, Hongdian Zhang, Yanli Qiu, Xueyan Bian, Xue Wang, Yue Li

**Affiliations:** ^1^ Department of Cardio‐pulmonary Functions Tianjin Medical University Cancer Institute and Hospital, National Clinical Research Center for Cancer, Key Laboratory of Cancer Prevention and therapy, Tianjin's Clinical Research Center for Cancer Tianjin China; ^2^ Department of Esophageal Cancer Tianjin Medical University Cancer Institute and Hospital, National Clinical Research Center for Cancer, Key Laboratory of Cancer Prevention and therapy, Key Laboratory of Digestive Cancer of Tianjin, Tianjin's Clinical Research Center for Cancer Tianjin China

**Keywords:** electrocardiogram, immune checkpoint inhibitors, QTc interval, severe ICI‐associated myocarditis, sinus tachycardia

## Abstract

**Objectives:**

Immune checkpoint inhibitor (ICI)‐associated myocarditis, particularly severe ICI‐associated myocarditis, has a high mortality rate. However, the predictive value of electrocardiogram (ECG) remains unclear. The present study aimed to evaluate the predictive value of clinical and electrocardiographic parameters for severe myocarditis.

**Methods:**

Clinical and electrocardiographic data of 73 cancer patients with ICI‐associated myocarditis were retrospectively collected. The severity of ICI‐associated myocarditis was graded using the NCCN guidelines for managing immunotherapy‐related toxicities. Myocarditis grades 1–2 and grades 3–4 were classified as mild and severe myocarditis, respectively. Logistic regression analysis was performed to analyze the predictive value of each parameter in predicting severe myocarditis.

**Results:**

Among the 73 patients with myocarditis, 20 (27.4%) patients had severe myocarditis. Compared with mild myocarditis group, sinus tachycardia (*p* = 0.001), QRS duration ≥110 ms (*p* = 0.001), prolonged QTc interval (*p* < 0.001), and bundle branch block (*p* = 0.007) at the time of myocarditis were more common in the severe myocarditis group. Logistic regression analysis revealed that sinus tachycardia (*p* = 0.028) and QTc interval prolongation (*p* = 0.007) were predictors of severe myocarditis. Whereas the predictive value of other electrocardiographic parameters was weak. Concurrent targeted therapy didn't increase the risk of severe myocarditis. A high NT‐proBNP level was associated with severe myocarditis.

**Conclusions:**

ECG at the onset of myocarditis manifested as sinus tachycardia and prolonged QTc interval predicted a high risk of severe myocarditis. Early detection of ECG abnormalities may faciliate early detection of severe ICI‐associated myocarditis.

## 1 INTRODUCTION

In recent years, immune checkpoint inhibitors (ICIs) as novel anticancer drugs have shown remarkable anti‐tumor efficacy and revolutionized tumor therapy.^1^ However, despite their better therapeutic effects, ICIs therapy could also lead to a series of immune‐related adverse events (irAE), particularly some lethal adverse effects such as myocarditis.^2^ ICI‐associated myocarditis can be classified into different grades according to the National Comprehensive Cancer Network (NCCN) guidelines for managing immunotherapy‐related toxicities. Patients with grade 3–4 myocarditis are at a high risk of occurrence of a major adverse cardiovascular event (MACE),^3^ which may negatively affect their prognosis. Mild myocarditis in the early stage of ICI treatment could rapidly develop into severe myocarditis, with a fatality rate up to 30%–50%.^4^ However, ICI‐associated myocarditis is usually asymptomatic and rare, and the incidence of this complication ranges from 0.09% to 1.1%.^2^, ^5^ Therefore, it is necessary for early diagnosis ICIs‐associated myocarditis and detection severe myocarditis to proceed immunotherapy process.

Several recent studies have reported the incidence, clinical characteristics, treatment, and outcomes of ICI‐associated myocarditis.^5^, ^6^, ^7^, ^8^ However, most of these studies focused on comparing patients with and without ICI‐associated myocarditis. Only a few studies have reported differences in clinical characteristics between patients with mild and severe myocarditis.^9^, ^10^ Electrocardiogram (ECG) is a simple, economical, and noninvasive test and can be chosen as the first‐line tool for detecting cardiovascular abnormalities in ICI users, particularly those with cardiac arrhythmias. Some studies reported the ECG changes in patients with ICI‐associated myocarditis, including low QRS voltage, pathological Q waves, and prolonged QRS interval.^11^, ^12^, ^13^, ^14^ However, very few investigations have reported differences in electrocardiographic manifestations between patients with mild and severe ICI‐associated myocarditis.^9^, ^15^ Therefore, to recognize severe myocarditis at an early stage and improve patent prognosis, there is an urgent requirement to determine the electrocardiographic characteristics of severe ICI‐associated myocarditis compared with mild ICI‐associated myocarditis.

Hence, in the present study, to better investigate the differences in the electrocardiographic characteristics of patients with mild and severe ICI‐associated myocarditis, we classified grades 1–2 and grades 3–4 ICI‐associated myocarditis, defined according to the NCCN guidelines for managing immunotherapy‐related toxicities as mild and severe myocarditis, respectively. We comprehensively compared the ECGs at the onset of myocarditis between patients with mild and severe myocarditis to determine the electrocardiographic characteristics that could predict severe myocarditis. We also analyzed the predictive significance of clinical characteristics and blood laboratory test results for patients with severe myocarditis. Our findings on differences in ECG changes between mild and severe myocarditis may facilitate early screening of severe myocarditis and enable the development of better monitoring strategies for ICIassociated myocarditis.

## 2 METHODS

### 2.1 Patients

This retrospective study included patients who received ICI treatment and were diagnosed to have ICI‐associated myocarditis at Tianjin Medical University Cancer Institute & Hospital from January 2020 to June 2023. The study was approved by the Ethics Committee of Tianjin Medical University Cancer Institute & Hospital. Written informed consent was waived because of the retrospective nature of the study.

The inclusion criteria were as follows: (1) pathological confirmation of malignancy; (2) received PD‐1 or PD‐L1 inhibitors treatment; (3) complete clinical and electrocardiographic data at the onset of myocarditis; and (4) diagnosis of ICI‐associated myocarditis according to the guidelines of the European Society of Cardiology,^16^ and the grading for ICI‐associated myocarditis was defined based on the NCCN Guidelines for managing of immunotherapy‐related toxicities^3^ as follows: Grade 1, asymptomatic, abnormal cardiac biomarkers (creatine kinase and troponin), abnormal ECG or physical findings (e.g., rub) consistent with pericarditis; Grade 2, mild symptoms or symptoms with moderate activity or exertion, abnormal screening tests: cardiac biomarkers (creatine kinase and troponin) and ECG; Grade 3, symptoms at rest or with minimal activity or exertion or new onset of symptoms; cardiac biomarkers (creatine kinase and troponin) > upper limit of the normal range; Grade 4, moderate to severe decompensation (worsening signs and symptoms), hemodynamic instability (hypotension/cardiomyopathy), cardiac biomarkers (creatine kinase and troponin levels) >3× upper limit of the normal range, life‐threatening; urgent intervention indicated. In the current study, to better investigate the association between electrocardiac parameters and the severity of ICI‐associated myocarditis, ICI‐associated myocarditis grades 1–2 were classified as mild myocarditis, whereas grades 3–4 were severe myocarditis. The grading for ICI‐associated myocarditis of each patients was defined by two clinicians, and a third clinician resolved any disagreements.

Exclusion criteria were as follows: (1) patients not receiving anti‐PD1/PD‐L1 immunotherapy; (2) patients with missing electrocardiographic data and laboratory test results; and (3) patients in whom the diagnosis of ICI‐associated myocarditis could not be definitely established; and (4) patients had history of myocarditis or who presenting clinical myocarditis before ICI treatment.

### 2.2 Clinical data collection

The following baseline characteristics were obtained retrospectively from the hospital's electronic medical record system: age, sex, smoking history, cardiovascular risk diseases, cancer type, tumor stage, occurrence of major adverse cardiac events (MACE), and immunotherapy‐related data (including types of ICIs, treatment cycles, treatment efficacy, and concurrent therapies). The results of the following blood laboratory tests were collected at the onset of myocarditis: serum creatine kinase (CK), creatine kinase MB (CK‐MB), cardiac troponin I (cTnI), N‐terminal pro‐B‐type natriuretic peptide (NT‐proBNP), and peripheral blood cells. Individuals were staged according to the 7th edition of the American Joint Committee on Cancer staging system. Neoadjuvant immunochemotherapy (NICT) responses were evaluated according to the Response Evaluation Criteria of Solid Tumors version 1.1(RECIST).^17^ The disease control rate (DCR) was evaluated, including complete remission (CR), partial remission (PR), stable disease (SD), and progressive disease (PD). MACE included cardiovascular death,^18^ cardiogenic shock,^19^ cardiac arrest,^20^ and hemodynamically significant complete heart block.^21^

### 2.3 ECG data collection and evaluation

All ECGs were recorded with standard 12‐lead placement at a standardization of 10 mm/mV and a paper speed of 25 mm/s. Baseline and at the onset of myocarditis ECGs data were collected for all patients. The primary variables included sinus arrhythmia (including normal sinus arrhythmia, sinus tachycardia, and sinus bradycardia), PR interval, QRS duration, QTc interval, atrial premature beat (APB), ventricular premature beat (VPB), ST‐T segment abnormalities, bundle branch block, and pathological Q wave.

Heart rate was defined as tachycardia or bradycardia according to the average rate of QRS complexes of >100/min or < 60/min, respectively. PR interval represents the time delay between atrial depolarization and ventricular depolarization; PR interval of >200 ms was considered prolonged. QRS duration was obtained from the first deflection of the QRS complex to the end of the QRS complex on the isoelectric line. QRS duration of >110 ms was considered prolonged.^22^ Bundle branch block, which included left/right bundle branch block, represents a disorder in the bundle branch conduction pathway, resulting in slow conduction.^22^ QT interval was calculated from the first deflection of the QRS complex to the end of the T wave on the isoelectric line. The QTc interval was calculated from the Bazett formula as follows: QTC=QT/√RR; (RR; heart rate). According to the standard criteria, a QTc interval of >450 ms in men and >460 ms in women was considered prolonged.^23^ ST‐T segment abnormalities included ST segment elevation (limb lead elevation >0.10 mV, thoracic lead >0.2 mV), ST segment depression (ST segment depression >0.05 mV for at least two leads), low T wave (limb lead <0.1 mV or chest lead <0.2 mV), bidirectional T wave (positive T wave followed by negative T wave), and inverted T wave (power wave inversion depth ≥0.1 mV).^24^ Two cardiologists independently reviewed each ECG, and a third cardiologist resolved any disagreements.

### 2.4 Statistical analysis

Continuous variables were expressed as mean ± SD or median, and categorical variables were expressed as percentages. Chi‐square test and Fisher's exact test were used to compare categorical variables, while Mann–Whitney *U* test was used to compare continuous variables between patients with mild and severe myocarditis. Logistic regression analysis was performed to assess the value of each parameter in predicting severe myocarditis. SPSS Statistics version 17.0 (SPSS, Inc., Chicago, IL) was used utilized for all statistical analyses. *p*‐values were corrected for multiple comparisons and a two‐sided *p*‐value of <0.05 was used to indicate statistical significance.

## 3 RESULTS

### 3.1 Patient and ECG characteristics

This retrospective study included 73 patients with cancer and diagnosed to have ICI‐associated myocarditis. The baseline clinical characteristics were summarized in Table 1. The median age was 63 years (range: 55–69 years), and 59 (80.8%) patients were males. The top three most common tumors were lung cancer (*N* = 34/73, 46.5%), gastrointestinal tumors (*N* = 15/73, 20.6%), and liver cancer (*N* = 10/73, 13.7%). Four (5.5%) patients had TNM stage II cancer, 21 (28.8%) patients had TNM stage III cancer, and 48 (65.7%) patients had stage IV cancer.

**TABLE 1:** Baseline clinical characteristics of the patients with immune checkpoint inhibitor‐associated myocarditis.

Characteristic	*N* (%)
Age (years)	63.0 (55.0–69.0)
Female	14 (19.2)
Primary cancer type	
Lung tumor	34 (46.5)
Gastrointestinal tumor	15 (20.6)
Liver cancer	10 (13.7)
Urinary tumor	7 (9.6)
Head and neck tumors	5 (6.9)
Others	2 (2.7)
TNM stage	
II	4 (5.5)
III	21 (28.8)
IV	48 (65.7)
Cardiovascular risk diseases	
Hypertension	28 (38.3)
Diabetes mellitus	9 (12.3)
Heart disease	7 (9.5)
None	29 (39.9)
Prior cardiovascular medications	
Aspirin	4 (5.5)
Beta‐blocker	5 (6.9)
ACE‐I or ARB	11 (15.1)
CCB	14 (19.2)
None	46 (63.0)
ICI types	
PD‐1 inhibitor	66.0 (90.4)
PD‐L1 inhibitor	7.0 (9.6)
Therapeutic cycles	2 (1.0–5.0)
Time from ICI medication to onset (days)	25.0 (20.0–37.5)
Initial presenting symptom	
Shortness of breath/Chest distress	17 (23.3)
Chest pain/Palpitations	6 (8.2)
Myasthenia/Fatigue	14 (19.1)
Blepharoptosis	9 (12.3)
Others	3 (4.1)
None	33 (45.2)
Other immune side effects	
Pituitary/adrenal axis disorder	11 (15.1)
Pneumonitis	7 (9.6)
Hepatitis	18 (24.7)
Dermatitis	1 (1.4)
Myositis	11 (15.1)
Neurological	1 (1.4)
Myocarditis grades	
Grades 1–2	53 (72.6)
Grades 3–4	20 (27.4)
Concurrent chemotherapy	46 (63.0)
Concurrent targeted therapy	26 (35.6)
Radiotherapy history	9 (12.3)
Immunotherapy efficacy	
CR	1 (1.4)
PR	14 (19.2)
SD	31 (42.4)
PD	16 (21.9)
Not evaluated	11 (15.1)
MACE	
No	64 (87.7)
Yes	9 (12.3)

Abbreviations: ACE‐I, angiotensin‐converting enzyme inhibitor; ARB, angiotensin receptor blocker; CCB, calcium channel blocker; CR, complete response; MACE, major adverse cardiovascular event; PD, progressive disease; PD‐1, programmed cell death 1; PD‐L1, programmed death ligand 1; PR, partial response; SD, stable disease; TNM stage, tumor node metastasis stage.

Overall, 66 (90.4%) and 7 (9.6%) patients were treated with PD‐1 and PD‐L1 inhibitors, respectively. Among those, 8 (11.0%), 17 (23.3%) and 48 (65.7%) patients received ICI therapy as neoadjuvant treatment, adjuvant treatment and metastatic palliative treatment, respectively. Based on the NCCN guidelines for managing of immunotherapy‐related toxicities, 53 (72.6%) patients were diagnosed to have grades 1–2 ICI‐associated myocarditis and were included in the mild myocarditis group, and 20 (27.4%) patients were diagnosed to have grades 3–4 ICI‐myocarditis and were included in the severe myocarditis group. All study patients completed ICIs therapy. Among the 73 patients, 1 (1.4%), 14 (19.2%), 31 (42.4%), and 16 (21.9%) patients exhibited CR, PR, SD, and PD, respectively, and the treatment efficacy could not be evaluated in 11 (15.1%) patients because of lack of imaging data.

Our results showed that ICI‐associated myocarditis occurred in the early treatment stage. The median number of days and immunotherapy cycles from the start of ICI treatment to myocarditis onset were 25.0 (20.0–37.5) days and 2 (1.0–5.0) cycles. The incidence of pneumonitis, liver injury, myositis, and pituitary/adrenal axis dysfunction was 9.6%, 24.7%, 15.1%, and 15.1%, respectively. Overall, the initial presenting symptom of ICI‐associated myocarditis was shortness of breath or chest distress in 17/73 (23.3%) patients, while 33/73 (45.2%) patients were asymptomatic. Among those, a total of 11/73 (15.1%) patients were diagnosed with myocarditis with myositis/myasthenia overlap syndrome, and 10/11 (90.9%) of patients were in the severe myocarditis group. Almost all patients in the severe myocarditis group showed initial presenting symptoms, and the most common symptom was precordial discomfort or chest tightness. Furthermore, nearly 60% of patients in the mild myocarditis group were asymptomatic. All patients in the severe myocarditis group received glucocorticoid treatment, and 45.0% of these patients received a combination of immunoglobulin and mycophenolate mofetil treatment. The overall incidence of MACE was 12.3% (9/73), and all these incidences occurred in patients from the severe myocarditis group.

We respectively collected and analyzed ECG data baseline and at the time of presentation with myocarditis. Overall, 32/73 (43.8%) patients had an abnormal ECG finding before ICI treatment, mainly including ST‐T segment abnormalities (17/73[23.3%]), sinus arrhythmia (10/73[13.7%]), QRS duration prolongation (6/73[8.2%]), and bundle branch block (5/73[6.8%]) Throughout ICI treatment, 53/73 (72.6%) of patients showed an abnormal ECG finding at the onset of myocarditis. ST‐T segment abnormality was the most common electrocardiographic abnormality (35/73 [47.9%]) at the onset of ICI‐myocarditis, followed by QTc interval prolongation (23/73[31.5%]), sinus arrhythmias (19/73 [26.0%]),  including sinus tachycardia (15/73 [20.5%]), sinus bradycardia (4/73 [5.5%]), QRS duration prolongation (14/73 [19.2%]), bundle branch block (13/73 [17.8%]), pathological Q wave (8/73 [11.0%]), VPB (7/73 [9.6%]), and PR interval prolongation (4/73 [5.5%]), and APB (3/73[4.1%]) (Table 2).

In the severe myocarditis group, 18/20 (90.0%) patients showed an abnormal ECG finding at the onset of myocarditis. The ECG of patients with severe myocarditis was various and non‐specific, which mainly included QTc interval prolongation (14/20 [70.0%]), sinus tachycardia (10/20 [50.0%]), ST‐T segment abnormalities (10/20 [50.0%]), QRS duration prolongation (9/20 [45.0%]), and bundle branch block (8/20 [40.0%]) (Table 2).

**TABLE 2:** Association between baseline and at the onset of ICI‐myocarditis ECG parameters with ICI–myocarditis severity

Baseline ECG parameters	Mild myocarditis, *N* (%) (*N* = 53)	Severe myocarditis, *N* (%) (*N* = 20)	*p‐*Value	ECG parameters at the onset of ICI‐myocarditis	Mild myocarditis, *N* (%) (*N* = 53)	Severe myocarditis, *N* (%) (*N* = 20)	*p‐*Value
*Sinus arrhythmia*			0.261	*Sinus arrhythmia*			**0.001**
Sinus tachycardia	4 (7.5)	4 (20.0)		Sinus tachycardia	5 (9.4)	10 (50.0)	
Sinus bradycardia	2 (3.8)	0 (0.0)		Sinus bradycardia	4 (7.5)	0 (0.0)	
Normal sinus rhythm	47 (88.7)	16 (80.0)		Normal sinus rhythm	44 (83.0)	10 (50.0)	
*ABP*			0.274	*ABP*			0.180
No	53 (100.0)	19 (95.0)		No	52 (98.1)	18 (90.0)	
Yes	0 (0.0)	1 (5.0)		Yes	1 (1.9)	2 (10.0)	
*VBP*			0.476	*VBP*			1.000
No	52 (98.1)	19 (95.0)		No	48 (90.6)	18 (90.0)	
Yes	1 (1.9)	1 (5.0)		Yes	5 (9.4)	2 (10.0)	
*ST‐T segment abnormalities*			1.000	*ST‐T segment abnormalities*			0.830
No	41 (77.4)	15 (75.0)		No	28 (52.8)	10 (50.0)	
Yes	12 (22.6)	5 (25.0)		Yes	25 (47.2)	10 (50.0)	
*QRS duration (ms)*			0.413	*QRS duration (ms)*			**0.001**
≥110	3 (5.7)	3 (15.0%)		≥110	5 (9.4)	9 (45.0)	
<110	50 (94.3)	17 (85.0)		<110	48 (90.6)	11 (55.0)	
*QTc interval (ms)*			0.105	*QTc interval (ms)*			**<0.001**
Normal QTc	52 (98.1)	17 (85.0)		Normal QTc	44 (83.0)	6 (30.0)	
Prolonged QTc	1 (1.9)	3 (15.0)		Prolonged QTc	9 (17.0)	14 (70.0)	
*PR interval (ms)*			1.000	*PR interval (ms)*			0.641
≥200	2 (3.8)	0 (0.0)		≥200	2 (3.8)	2 (10.0)	
<200	51 (96.2)	20 (100.0)		<200	51 (96.2)	18 (90.0)	
*Bundle branch block*			0.240	*Bundle branch block*			**0.007**
No	51 (96.2)	17 (85.0)		No	48 (90.6)	12 (60.0)	
Yes	2 (3.8)	3 (15.0)		Yes	5 (9.4)	8 (40.0)	
*Pathological Q wave*			0.476	*Pathological Q wave*			0.272
No	52 (98.1)	19 (95.0)		No	49 (92.5)	16 (80.0)	
Yes	1 (1.9)	1 (5.0)		Yes	4 (7.5)	4 (20.0)	

Abbreviations: ABP, atrial premature beat; ECG, electrocardiogram; VBP, ventricular premature beat.

### 3.2 Association of electrocardiographic and clinical characteristics with the severity of ICI‐associated myocarditis

The clinical characteristics were compared between the mild and severe myocarditis groups to identify indicators for severe myocarditis (Table 3). We found that patients' characteristics, including gender, age, smoking and drinking history, diabetes, hypertension, heart disease, and prior cardiovascular medications were not associated with myocarditis severity. Additionally, immunotherapy‐related parameters, including therapeutic cycles, ICI drugs, concurrent chemotherapy, and radiotherapy history, were also not significantly associated with myocarditis severity (all *p*‐values >0.05). However, only concurrent targeted therapy was inversely associated with severe myocarditis (*p* = 0.047).

**TABLE 3:** Differences in clinical features between patients with mild and severe ICI‐associated myocarditis.

	Total cohort, *N* (%) (*N* = 73)	Mild myocarditis, *N* (%) (*N* = 53)	Severe myocarditis, *N* (%) (*N* = 20)	*p‐*Value
Gender				
Male	59 (80.8)	44 (83.0)	15 (75.0)	0.509
Female	14 (19.2)	9 (17.0)	5 (25.0)	
Age (years)		61.7 ± 10.1	63.4 ± 8.9	0.524
Smoking history				
No	25 (34.2)	19 (35.8)	6 (30.0)	0.784
Yes	48 (65.8)	34 (64.2)	14 (70.0)	
Drinking history				
No	49 (67.1)	37 (69.8)	12 (60.0)	0.577
Yes	24 (32.9)	16 (30.2)	8 (40.0)	
Diabetes				
No	64 (87.7)	47 (88.7)	17 (85.0)	0.978
Yes	9 (12.3)	6 (11.3)	3 (15.0)	
Hypertension				
No	45 (61.6)	31 (58.5)	14 (70.0)	0.428
Yes	28 (38.4)	22 (41.5)	6 (30.0)	
Heart disease				
No	66 (90.4)	49 (92.5)	17 (85.0)	0.604
Yes	7 (9.6)	4 (7.5)	3 (15.0)	
Prior cardiovascular medications				
No	46 (63.0)	31 (58.5)	15 (75.0)	0.278
Yes	27 (37.0)	22 (41.5)	5 (25.0)	
Immunotherapy‐related information				
Therapeutic cycles		3 (1–5)	2 (1–5.5)	0.330
Immunotherapy types				
PD‐1 inhibitor	66 (90.4)	48 (90.6)	18 (90.0)	1.000
PD‐L1 inhibitor	7 (9.6)	5 (9.4)	2 (10.0)	
Concurrent chemotherapy				
No	27 (37.0)	20 (37.7)	7 (35.0)	1.000
Yes	46 (63.0)	33 (62.3)	13 (65.0)	
Concurrent targeted therapy				
No	47 (64.4)	30 (56.6)	17 (85.0)	**0.047**
Yes	26 (35.6)	23 (43.3)	3 (15.0)	
Radiotherapy history				
No	64 (87.7)	49 (92.5)	15 (75.0)	0.104
Yes	9 (12.3)	4 (7.5)	5 (25.0)	
Blood laboratory tests				
cTnI (pg/ml)	246.7 (87.2–1114.7)	168.1 (67.8–391.3)	1284.5 (157.5–8218.8)	**0.003**
NT‐proBNP (pg/ml)	12.5 (5.0–39.8)	7.0 (5.0–33.0)	33.6 (11.3–89.4)	**0.002**
CK (ug/L)	224.0 (79.5–1169.0)	185.0 (70.5–895.8)	661.0 (195.0–3470.0)	**0.009**
CK‐MB (ug/L)	8.6 (3.5–37.8)	6.3 (2.9–20.3)	44.5 (5.1–142.2)	**0.006**

Abbreviations: CK, Creatine kinase; CK‐MB, Creatine kinase‐MB; cTnI, cardiac Troponin I; NT‐proBNP, pro‐B type natriuretic peptide.

We further analyzed differences in serum cardiac biomarkers at the onset of ICI‐associated myocarditis between the two groups. The median values of cTnI, NT‐proBNP, CK, and CK‐MB were higher in the severe myocarditis group than in the mild myocarditis group (median values: 1284.5 [157.5–8218.8] vs. 168.1 [67.8–391.3], *p* = 0.003; 33.6 [11.3–89.4] vs. 7.0 [5.0–33.0], *p* = 0.002; 661.0 [195.0–3470.0] vs. 185.0 [70.5–895.8], *p* = 0.009; and 44.5 [5.1–142.2] vs. 6.3 [2.9–20.3], *p* = 0.006, respectively). We then examined the association of electrocardiographic parameters with the severity of ICI‐associated myocarditis. As shown in Table 2, the pre‐ICI baseline sinus arrythmia, APB, VPB, ST‐T segment abnormalities, QRS duration, QTc interval, PR interval, bundle branch block and pathological Q wave were similar between mild and severe myocarditis groups (all *p*‐values >0.05). A similar testing strategy was adopted for analyzing the association of electrocardiographic parameters at the onset of ICI‐associated myocarditis with severe myocarditis. The following on‐ICI electrocardiographic abnormalities were more commonly observed in the severe myocarditis group than in the mild myocarditis group: sinus tachycardia (*p* = 0.001), QRS duration ≥110 ms (*p* = 0.001), prolonged QTc interval (*p* < 0.001), and bundle branch block (*p* = 0.007). Whereas, no differences were observed in the incidence of APB, VPB, ST‐T segment abnormalities, PR interval prolongation, and pathological Q wave (all *p‐*values >0.05).

To investigate the effects of the above‐mentioned four electrocardiographic abnormalities at the onset of ICI‐associated myocarditis to predict the severe of ICI‐associated myocarditis, we further analyzed the differences of electrocardiographic parameters between baseline and at the onset of ICI‐associated myocarditis. With this approach, the percentage of sinus arrhythmia (*p* = 0.008), QRS duration (*p* = 0.011), the QTc interval (*p* = 0.008) and bundle branch block (*p* < 0.001) at the onset of myocarditis were increased when compared to baseline electrocardiographic parameters (Table S1). We further explored if above ECG abnormalities at the onset of ICI‐associated myocarditis were associated with MACE. In all nine ICI‐associated myocarditis patients with MACE, 5/9 (55.6%) patients harbored with sinus tachycardia, 3/9 (33.3%) patients harbored with prolonged QRS duration, 5/9 (55.6%) patients harbored with prolonged QTc interval, and none of the patients harbored with bundle branch block. We found that only sinus tachycardia (*p* = 0.031) at the onset of ICI‐associated myocarditis was associated with MACE, which prolonged QRS duration (*p* = 0.484), prolonged QTc interval (*p* = 0.202), and bundle branch block (*p* = 1.000) did not show a clear relationship with MACE (Table S2).

### 3.3 Predictive value of ECG parameters for severe ICI‐associated myocarditis

To assess the predictive ability of the clinical and electrocardiographic characteristics, we conducted a logistic regression analysis based on the results of univariate analyses to determine the appropriate indicators that could predict the severity of ICI‐associated myocarditis. We found that concurrent targeted therapy, sinus arrhythmia, QTc interval prolongation, and high NT‐proBNP level were predictors of severe myocarditis. The mild and severe myocarditis groups showed significant differences in concurrent targeted therapy (hazard ratio [HR]: 0.058, 95% confidence interval [CI]: 0.006–0.523, *p* = 0.011), sinus arrhythmia (HR: 3.257, 95% CI: 1.137–9.325, *p* = 0.028), QTc interval prolongation (HR: 14.185, 95% CI: 2.046–98.339, *p* = 0.007), and high NT‐proBNP Level (HR: 10.743, 95% CI: 1.382–83.5204, *p* = 0.023) (Table 4).

**TABLE 4:** Logistic regression analysis to determine the predictive value of clinical and electrocardiographic characteristics for ICI‐associated myocarditis severity.

Variables	HR	95% CI	*p* value
Concurrent targeted therapy	0.058	0.006–0.523	**0.011**
Sinus arrhythmia	3.257	1.137–9.325	**0.028**
QRS duration	8.702	0.267–283.453	0.223
QTc interval	14.185	2.046–98.339	**0.007**
Bundle branch block	0.254	0.007–9.943	0.464
cTnI	0.724	0.105–4.991	0.743
NT‐proBNP	10.743	1.382–83.520	**0.023**
CK	0.611	0.041–9.024	0.720
CK‐Mb	3.424	0.223–52.645	0.377

Abbreviations: cTnI, cardiac Troponin I; CI, confidence interval; CK, Creatine kinase; CK‐MB, Creatine kinase‐MB; HR, hazard ratio; NT‐proBNP, pro‐B type natriuretic peptide.

In contrast, the following variables could not predict the occurrence of severe ICI‐associated myocarditis: QRS interval ≥ 110 ms (HR: 8.702, 95% CI: 0.267–283.453, *p* = 0.223), bundle branch block (HR: 0.254, 95% CI: 0.007–9.943, *p* = 0.464), serum cTnI elevation (HR: 0.724, 95% CI: 0.105–4.991, *p* = 0.743), elevated serum CK level (HR: 0.611, 95% CI: 0.041–9.024, *p* = 0.720), and elevated serum CK‐MB value (HR: 3.424, 95% CI: 0.223–52.645, *p* = 0.377) (Table 4).

## 4 DISCUSSION

Although ICI‐associated myocarditis is a severe irAE, patients with this complication usually do not exhibit specific clinical manifestations. Furthermore, severe ICI‐associated myocarditis often shows a violent course and may be at a high risk of occurrence of MACE, such as acute heart failure and cardiogenic shock.^21^ Therefore, it is essential to promptly identify cases of severe ICI‐associated myocarditis and implement effective therapies. Thus far, some serum or echocardiographic markers have been reported to predict severe ICI‐associated myocarditis.^9^, ^10^ However, few studies were focused on comparing patients with mild and severe myocarditis and most studies explored the predictive value of fewer electrocardiographic parameters.

ECG, a simple and noninvasive test, could enable early detection of ICI‐associated myocarditis before the occurrence of clinical manifestations and changes in serum cardiac markers or left ventricular ejection fraction. However, to date, few studies have reported differences in electrocardiographic characteristics between patients with mild and severe myocarditis and have evaluated the predictive ability of electrocardiographic parameters for stratifying the severity of ICI‐associated myocarditis. In this paper, we present novel findings describing the predictive value of comprehensive electrocardiographic parameters on predicting the severity of ICI‐associated myocarditis and identified the relevant predictors of severe myocarditis. The comparison of the clinical and electrocardiographic characteristics of patients with severe and mild ICI‐associated myocarditis yielded several important results. First, severe ICI‐associated myocarditis occurred in the early stages of treatment and was accompanied by initial presenting symptoms, the most common of which were precordial discomfort or chest tightness. Second, almost all patients in the severe myocarditis group had elevated levels of cardiac biomarkers, and a high NT‐proBNP level was one of the predictors of severe myocarditis. Third, the severe myocarditis group showed a high incidence of MACE. Fourth and most critically, almost all patients in the severe myocarditis group showed abnormalities in their ECG results at the onset of myocarditis. Sinus arrhythmia (especially sinus tachycardia) and QTc interval prolongation at the onset of ICI‐myocarditis but not baseline ECG parameters were the predictors of severe myocarditis in this group.

Tang et al.^9^ retrospectively analyzed the clinical characteristics of patients with ICI‐associated myocarditis and reported several predictors of severe ICI‐associated myocarditis. However, they did not found that ECG abnormalities were associated with the severity of ICI‐associated myocarditis. In contrast, our results demonstrated that ECG abnormalities (mainly sinus tachycardia and QTc interval prolongation) were associated with myocarditis severity. This inconsistency could be explained by the differences in the criteria used for grading myocarditis severity and the fewer ECG parameters included in the previous study. Our study findings also did not completely agree with the results of Zlotoff et al.,^13^ who reported that the QRS duration, but not QTc interval, was increased at myocarditis onset as compared to the pre‐myocarditis baseline value. However, in a preliminary analysis, we found an initial increase in the QRS duration of patients in the severe myocarditis group as compared to that in the mild myocarditis group patients; this difference disappeared once sinus tachycardia and QTc interval prolongation were included in the logistic regression analysis. A noteworthy finding is our study population was different from the cohort in the study of Zlotoff et al.^13^ These authors focused on comparing the clinical characteristics of patients with and without ICI‐associated myocarditis, while our study mainly analyzed the differences in ECG abnormalities between mild and severe myocarditis. Moreover, our study showed findings similar to those of the study of Liu et al., in which they reported ECG manifestations of sinus tachycardia, ST‐T segment changes, and prolonged QTc interval in patients with severe ICI‐associated myocarditis.^15^ The specific electrocardiographic mechanisms of severe ICI‐associated myocarditis are poorly understood. Histologically, patients with ICI‐associated myocarditis show dense acute lymphocytic infiltration in the myocardium.^25^ Previous studies have shown an increase in effector cytotoxic CD8+ cells in mice models of PD‐1 blockade.^26^ These expanded CD8+ cells, along with the upregulated levels of the chemokines CCL5/CCL4/CCL4L2, may affect both the myocardium and the electrical conduction system.^27^ Therefore, the increase in sinus heart rate and prolongation in QT interval in patients with severe ICI‐associated myocarditis may be mediated by lymphocyte infiltration, which may affect both the sinus node and the conduction system.

ICI‐associated myocarditis is known to have a higher risk of adverse events as compared to other irAEs.^2^ The present study also showed that patients in the severe myocarditis group were more likely to develop MACE. Sinus arrhythmia (especially sinus tachycardia) at the onset of ICI‐associated myocarditis was associated with MACE. However, other electrocardiographic parameters and serum cardiac biomarkers were not associated with MACE in this study. These important results need to be interpreted carefully. New sinus tachycardia may early suggest ICI‐related electrophysiological and functional myocardial injury cardiotoxicity and a higher risk of developing malignant arrhythmia and MACE. Treatment with high‐dose corticosteroids, gamma globulins, and immunosuppressants can improve outcome of severe myocarditis.^28^ In our study cohort, all patients in the severe myocarditis group received high‐dose corticosteroid therapy, and nearly 60% of the patients received corticosteroids in combination with gamma globulins and/or immunosuppressants. These findings emphasized the importance of early identification and diagnosis of severe myocarditis in patients receiving ICIs.

In our study, VPB/APB, ST‐T segment abnormalities, PR interval, and pathological Q wave were not associated with the severity of myocarditis in preliminary analysis. Moreover, in the logistic regression analysis, QRS duration and bundle branch block showed no differences between the mild and severe myocarditis groups. Patients with ICI‐associated myocarditis may have new changes in ECG as compared to the baseline findings, manifested as arrhythmias, conduction block, ST‐T segment abnormalities, pathological Q waves, and low QRS voltage at multiple leads.^11^ However, the above‐ mentioned electrocardiographic characteristics of ICI‐associated myocarditis have been rarely reported as predictors of severe myocarditis as compared to the predictors for mild myocarditis. Although ICI‐associated myocarditis is highly arrhythmogenic, the specific electrocardiographic manifestations are poorly understood. We also showed that VPB/APB was not related to severe ICI‐associated myocarditis. Pathological Q wave is a manifestation of electrically inert myocardium. In ICI‐associated myocarditis, immune cell infiltrates lead to cardiomyocyte necrosis and electrical inactivity.^29^ However, it is difficult to differentiate pathological Q waves of ICI‐associated myocarditis from myocardial infarction (MI) and cardiomyopathy. ST‐T represents the process of ventricular muscle repolarization. Various factors can lead to changes in ST‐T in the ECG, including sinus tachycardia, cardiac conduction disorder, or nonspecific ST changes. Therefore, in view of the different centers show variations in the results of ECG parameters related to ICI‐associated myocarditis, further research is required to determine which electrocardiographic parameters are appropriate to stratify the severity of ICI‐associated myocarditis.

In recent years, several clinical trials have evaluated the effects of PD‐1/PDL1 and/or the combination of chemotherapy, radiotherapy, and targeted therapy from different perspectives.^30^ Therefore, we examined whether ICI treatment cycles, ICI types, and concurrent systematic therapies (including chemotherapy, radiotherapy, and targeted therapy) were high‐risk factors for severe ICI‐associated myocarditis. We found that, except for concurrent targeted therapy, ICI treatment cycles, ICI types, concurrent chemotherapy, and radiotherapy history were not related to myocarditis severity. Hence, for patients in the early or late stages of immunotherapy, those receiving different types of ICI, and those undergoing concurrent chemotherapy and radiotherapy, clinicians should equally focus on the occurrence of severe myocarditis. Tang et al.^9^ also noted that ICI treatment cycles, ICI types, and concurrent systematic therapies did not increase the risk of severe ICI‐associated myocarditis. Additionally, a noteworthy finding of our study was that concurrent targeted therapy did not increase myocarditis severity, but it reduced the risk of severe ICI‐associated myocarditis. There is currently no consensus regarding whether the combination of systemic therapy increases the risk of cardiovascular events during ICI treatment. Wu et al. reported that the combination of ICI drugs and vascular endothelial growth factor receptor inhibitors had an increased risk of cardiovascular adverse events.^31^ Yet, a meta‐analysis by Agostinetto et al. clarified that the use of ICI as a single or combination systemic therapy did not increase the risk of cardiotoxicity.^32^ Given this inconsistency in views regarding on the risk of cardiovascular events following systemic therapy combined with ICIs and the small sample size of our study, these findings should be interpreted with caution. Hence, larger and prospective studies of ICI‐based combinations are required to further confirm these findings.

Cardiac biomarkers, including troponin, CK, CK‐MB, and BNP/NT‐proBNP, have been reported to indicate acute coronary syndromes and heart failure.^33^ Among these biomarkers, elevated cTnI and BNP/NT‐proBNP levels were shown to be associated with increased mortality of cancer patients and to predict the occurrence of ICI‐associated cardiotoxicities, including myocarditis.^34^ TnI is the primary biomarker for the diagnosis of acute MI. Puzanov I et al. reported that elevated cTnI, CK, and CK‐MB were the predictors of myocarditis and mortality. However, BNP did not show a correlation with progression to severe myocarditis.^35^ Our preliminary analysis revealed that cTnI, CK, CK‐MB, and NT‐proBNP levels were related to severe ICI‐associated myocarditis. However, in the subsequent multivariate analysis, only NT‐proBNP was correlated with an increased risk of severe ICI‐associated myocarditis. These results are in contrast with current ICI myocarditis diagnostic guidelines.^36^ Meanwhile, our findings were consistent with those of Xu et al., who reported that advanced non‐small cell lung cancer patients with severe myocarditis exhibited higher BNP levels than those with mild myocarditis.^37^ NT‐proBNP is synthesized and secreted by cardiomyocytes, which are markers of cardiac wall stress and heart failure criterion.^38^ Therefore, it was speculated that patients with severe ICI‐associated myocarditis had a risk of developing heart failure. Because of the small sample size of study, the different cTnI assays and timing of sample procurement, the predictive value of NT‐proBNP not cTnI in severe myocarditis cannot be ruled out. Further investigations are required to confirm the utility of BNP in predicting severe ICI‐associated myocarditis. Nonetheless, we still support the idea that early and serial measurements of cTnI, CK, CK‐MB, and NT‐proBNP could be useful for the early diagnosis of severe ICI‐associated myocarditis and improving prognosis through appropriate treatment interventions.

Considering several limitations of the present study, our results should be interpreted with caution. First, this was a retrospective, single‐center study with a small sample size; hence, there is an inevitable selection bias associated with our findings. Second, this study enrolled patients with heterogeneous tumor types and different treatment regimens in combination with ICI. Thus, more homogenous conclusions could not be derived from our results. Finally, echocardiography data were missing for some patients during the follow‐up period, which likely enriched for patients with ICI‐associated myocarditis. Despite these weaknesses, our study is the first to report the comprehensive differences in the electrocardiographic characteristics of patients with mild and severe ICI‐associated myocarditis. Large prospective and multicenter studies are required in the future to validate the findings of the present study.

## 5 CONCLUSIONS

Severe ICI‐associated myocarditis has a high risk of mortality; hence, it is critical to detect this complication early to improve patient prognosis. The present study comprehensively investigated the relevance of ECG abnormalities in predicting the severity of ICI‐associated myocarditis. We found that ECG of patients with severe ICI‐associated myocarditis manifested as sinus tachycardia and prolonged QTc interval. Concurrent targeted therapy did not increase the risk of severe myocarditis. Furthermore, a correlation was observed between higher serum NT‐proBNP levels and the severity of ICI‐associated myocarditis. During ICI treatment, regular ECG monitoring is crucial for the early identification of severe ICI‐associated myocarditis, as this could enable the prompt implementation of therapeutic interventions for patients with high‐risk features.

## AUTHOR CONTRIBUTIONS

**Yongyin Gao:** Data curation (lead); formal analysis (equal); methodology (lead); writing – original draft (lead); writing – review and editing (lead). **Hongdian Zhang:** Formal analysis (equal); methodology (equal). **Yanli Qiu:** Formal analysis (equal). **Xueyan Bian:** Formal analysis (equal). **Xue Wang:** Validation (equal). **Yue Li:** Conceptualization (lead).

## FUNDING INFORMATION

This work was not funded by any grant.

## CONFLICT OF INTEREST STATEMENT

The authors declared no conflict of interest.

## ETHICS STATEMENT

The data collection and processing protocols were performed in accordance with the Declaration of Helsinki, approved by the institutional ethics committee (Ethics Committee of Tianjin Medical University Cancer Institute & Hospital).

## Supporting information


Table S1.

Table S2.

## Data Availability

All analyzed data are included in this published article. The original data used in the current study are available from the corresponding author on reasonable request.
