# Supplementary material for: Early identification of severe immune checkpoint inhibitor associated myocarditis: From an electrocardiographic perspective
Source: Cancer Med. 2024 Jul 31;13(15):e7460. doi: 10.1002/cam4.7460 (PMC11289619; doi:10.1002/cam4.7460)
Supplement: Supplementary file 1 — Table S1. [file CAM4-13-e7460-s001.docx]

**Supplementary Table1.** Comparisons of baseline and at the onset of ICI-myocarditis ECG parameters as predictors of ICI-associated myocarditis severity

|  | **Baseline ECG parameters, N (%)**  **(N = 73)** | **at the onset of ICI-myocarditis ECG parameters, N (%)**  **(N = 73)** | ***P-*value** |
| --- | --- | --- | --- |
| Sinus arrhythmia  Sinus tachycardia  Sinus bradycardia  Normal sinus rhythm | 8 (11.0)  2 (2.7)  63 (86.3) | 15 (20.5)  4 (5.5)  54 (74.0) | **0.008** |
| QRS duration(ms)  ≥110  <110 | 6 (8.2)  67 (91.8) | 14 (19.2)  59(80.8) | **0.011** |
| QTc interval(ms)  Normal QTc  Prolonged QTc | 69 (94.5)  4 (5.5) | 50 (68.5)  23 (31.5) | **0.008** |
| Bundle branch block  No  Yes | 68 (93.2)  5 (6.8) | 60 (82.2)  13 (17.8) | **<0.001** |
