# Supplementary material for: Early identification of severe immune checkpoint inhibitor associated myocarditis: From an electrocardiographic perspective
Source: Cancer Med. 2024 Jul 31;13(15):e7460. doi: 10.1002/cam4.7460 (PMC11289619; doi:10.1002/cam4.7460)
Supplement: Supplementary file 2 — Table S2. [file CAM4-13-e7460-s002.docx]

**Supplementary Table 2.** Association between ECG parameters predicting severe ICI-associated myocarditis with major adverse cardiovascular event (MACE)

|  | **MACE (−), N (%)**  **(N = 64)** | **MACE (+), N (%)**  **(N = 9)** | ***P-*value** |
| --- | --- | --- | --- |
| Sinus arrhythmia  Sinus tachycardia  Sinus bradycardia  Normal sinus rhythm | 10 (15.6)  4 (6.3)  50 (78.1) | 5 (55.6)  0 (0.0)  4 (44.4) | **0.031** |
| QRS duration(ms)  ≥110  <110 | 11 (17.2)  53 (82.8) | 3 (33.3)  6 (66.7) | 0.484 |
| QTc interval(ms)  Normal QTc  Prolonged QTc | 46 (71.9)  18 (28.1) | 4 (44.4)  5 (55.6) | 0.202 |
| Bundle branch block  No  Yes | 53 (82.8)  11 (17.2) | 7 (77.8)  2 (22.2) | 1.000 |
